# Psychotherapy initiation is associated with discontinuation of psychotropic medications without dose escalation: a ten-year real-world cohort study (2014-2024)

**DOI:** 10.3389/fpsyt.2026.1841866

**Published:** 2026-06-22

**Authors:** Teresa Dolores Pomares Carreres, Guillermo Martínez Conesa, Francisco Navarrete

**Affiliations:** 1 Department of Mental Health, Marina Baixa Hospital, Villajoyosa, Alicante, Spain; 2 Department of Preventive Medicine and Public Health, Faculty of Medicine, University of Murcia, Murcia, Spain; 3 Instituto de Neurociencias, Universidad Miguel Hernández-CSIC, San Juan de Alicante, Alicante, Spain; 4 Red de Investigación en Atención Primaria de Adicciones, Instituto de Salud Carlos III, MICINN and FEDER, Madrid, Spain; 5 Instituto de Investigación Sanitaria y Biomédica de Alicante (ISABIAL), Alicante, Spain

**Keywords:** antidepressants, benzodiazepines, cohort study, pharmacoepidemiology, psychotherapy, psychotropic medication reduction, psychotropic polypharmacy, real-world evidence

## Abstract

**Background:**

Increasing psychotropic prescribing has raised concerns about long-term safety and regimen complexity in mental health care. Although psychotherapy is an established treatment, its role in medication optimization and psychotropic medication reduction in real-world practice across patient subgroups remains insufficiently characterized.

**Objective:**

To evaluate whether initiation of psychotherapy is associated with short-term changes in psychotropic medication exposure and regimen complexity, and to examine differences by sex, age, and diagnostic category. Methods: A retrospective cohort study was conducted using anonymized pharmacy dispensing data from the Mental Health Service of Hospital Marina Baixa (Alicante, Spain) between 2014 and 2024. Patients with at least one active prescription for a benzodiazepine or antidepressant within 90 days before psychotherapy initiation were included. Psychotropic exposure was compared in symmetric 90-day pre- and post-therapy windows using number of active agents, total Defined Daily Doses, and prevalence of benzodiazepine and antidepressant use, with stratified analyses by sex, age group, and diagnosis.

**Results:**

The cohort comprised 86,502 patients and 20.76 million dispensations. The median number of psychotropic medications decreased from 5 to 2 (p < 0.001), while total dose remained stable (median Defined Daily Dose ≈ 21.7). Benzodiazepine use declined from 87.6% to 67.5% and antidepressant use from 81.8% to 68.8% (both p < 0.001). Men were more likely than women to discontinue benzodiazepines (odds ratio 1.27, 95% confidence interval 1.13–1.43), and simplification increased with age (median reduction −1 in <18 years to −4 in ≥65 years). The largest benzodiazepine reductions occurred in depressive, personality, and episodic mood disorders (−23 to −27 percentage points).

**Conclusions:**

In routine public mental health care, psychotherapy initiation is associated with substantial simplification of psychotropic treatment regimens without increasing overall medication dose, supporting a potential role in facilitating rational medication simplification.

## 1 Introduction

Psychotropic medication use has expanded steadily across Europe during the past two decades, reflecting both improved access to treatment and growing medicalization of psychological distress (1–3). Spain has exhibited one of the highest prescription rates for benzodiazepines and antidepressants in the region, and post-pandemic data reveal further acceleration of these trends (4–6). This escalation raises concerns about polypharmacy, adverse drug interactions, and dependence potential (7–9).

Efforts to rationalize prescribing have traditionally relied on pharmacological deprescribing programs, medication reviews, or clinician education. Although conceptually sound, these interventions often produce modest and short-lived effects (10–12). Sustained reduction in psychotropic exposure may require complementary behavioral strategies capable of reshaping patient coping patterns and prescriber decision-making processes.

Psychotherapy, particularly cognitive-behavioral approaches, has well-documented efficacy for anxiety and mood disorders (13–15). Beyond symptom relief, psychotherapy fosters self-efficacy, adaptive emotional regulation, and cognitive restructuring, mechanisms that can enable both clinicians and patients to reassess the need for continued pharmacological support. Engagement in structured therapy may also provide an alternative therapeutic identity that reduces reliance on medication as a coping resource (16–18).

Despite this theoretical rationale, empirical data quantifying psychotherapy’s deprescribing potential in real-world environments remain scarce. Most previous studies have been limited by small sample sizes, self-reported medication use, or short observation windows (19–21). Few investigations have taken advantage of dispensing databases, which objectively measure medication exposure through Defined Daily Doses (DDD) and number of active molecules dispensed. Analyzing dispensing data before and after psychotherapy initiation offers a powerful naturalistic design to identify potential shifts in medication utilization temporally aligned with psychological intervention.

The current study addresses this evidence gap by analyzing a decade of real-world dispensing data from a regional public mental-health network. It aims to evaluate whether initiating psychotherapy is associated with short-term changes in psychotropic medication use and treatment complexity, focusing on DDD, active molecule counts, and prevalence of benzodiazepine and antidepressant exposure.

This study hypothesizes that psychotherapy initiation would be linked to a clinically relevant reduction in the number of concurrent psychotropic agents and in the prevalence of benzodiazepine and antidepressant use, without an increase in total DDD. Demonstrating such an association could provide empirical support for considering the integration of psychotherapy into psychotropic medication reduction-oriented care frameworks. Moreover, it would contribute to a growing understanding of how non-pharmacological interventions can improve medication safety, reduce unnecessary polypharmacy, and foster sustainable prescribing practices within public health systems.

In a broader sense, this research aligns with the international agenda promoting rational psychotropic use and person-centered care. By providing robust pharmacoepidemiological evidence from routine clinical practice, it may inform policies aimed at balancing pharmacological and psychological approaches to mental-health treatment, guiding both prescribers and health authorities towards safer, evidence-based prescribing cultures.

## 2 Materials and methods

### 2.1 Study design

This study employed a retrospective, observational, within-subject cohort design based on anonymized pharmacy dispensing data. The design was selected to evaluate temporal changes in psychotropic drug exposure associated with the initiation of psychotherapy under real-world conditions. A within-subject pre–post comparison was used to minimize between-person confounding and to capture naturalistic medication dynamics across a large population.

The analytical framework followed established pharmacoepidemiological standards for assessing medication utilization and medication utilization indicators in administrative health data. The guiding principle was to determine whether the beginning of psychotherapy corresponded with measurable simplification of psychotropic treatment regimens, independent of diagnostic or sociodemographic variables.

### 2.2 Setting and data source

Dispensing information was extracted from the Hospital Marina Baixa Mental Health Department electronic pharmacy registry, which captures every reimbursed dispensation in community pharmacies within the corresponding health district of the Valencian Health Service (Spain). This database contains longitudinal records of all prescriptions filled under the public healthcare system, covering a population of approximately 400–000 inhabitants.

Each record includes the following variables: anonymized patient identifier, date of dispensation, Anatomical Therapeutic Chemical (ATC) code, product name, pharmaceutical form, strength, quantity, and prescriber category. Duplicates and administrative reversals were excluded through automated data-cleaning scripts. The system operates under mandatory reporting, ensuring near-complete capture of reimbursed prescriptions.

The dataset is suitable for pharmacoepidemiological research because it represents dispensed medications rather than prescriptions issued, thus reflecting actual patient-level exposure. This distinction reduces bias associated with non-adherence or non-collection of prescribed medicines.

### 2.3 Study period and data extraction

The observation window spanned January 1, 2014, to December 31, 2024, providing ten full years of data. For each eligible patient, records were extracted for a 180-day window centered on the date of psychotherapy initiation (“day 0”), encompassing the 90 days before and after the first session.

Extraction was performed via a structured query written in SQL and validated independently by two analysts. Dispensing events were aggregated per molecule and patient, yielding daily exposure variables expressed in Defined Daily Doses (DDD).

To ensure reproducibility, all scripts were version-controlled and archived within the institutional secure research environment.

### 2.4 Study population

Eligible individuals were those with at least one psychotherapy session recorded in the institutional mental-health information system during the study period, at least one active dispensation of a benzodiazepine (BZD) or antidepressant (AD) within the 90 days preceding day 0, and continuous pharmacy coverage, defined as ≥1 dispensation of any psychotropic drug in each of the two 90-day windows. Patients with incomplete symmetric data (i.e., missing dispensing information in either the pre- or post-therapy window) were excluded. Individuals receiving inpatient psychiatric care or specialized institutional treatment during the observation periods were also excluded to avoid bias from hospital-supplied medication. The final analytic cohort comprised 86,502 unique anonymized patients and 20,759,923 dispensing records.

Sociodemographic variables included sex and age (continuous and categorized as <18, 18–39, 40–64, and ≥65 years). Clinical information was based on the primary ICD-9-CM diagnosis, grouped into categories relevant to psychotropic use: mood disorders (296), anxiety disorders (300), personality disorders (301), acute stress reactions (308), adjustment disorders (309), unspecified depressive disorders (311), and emotional disorders of childhood and adolescence (313).

Diagnostic categories were grouped according to clinically related ICD-9 classifications to facilitate interpretation and ensure sufficient sample sizes within each category. Primary diagnosis was defined as the main recorded psychiatric diagnosis closest to the psychotherapy initiation date (day 0). Because diagnostic coding may vary over time in routine clinical practice, diagnoses were treated as pragmatic clinical groupings rather than fixed longitudinal disease classifications.

To evaluate potential selection bias, additional analyses were conducted comparing included and excluded patients (those lacking complete pre–post data) in terms of age, sex, diagnostic category, and baseline medication burden.

### 2.5 Exposure definition

The exposure was the initiation of psychotherapy, operationalized as the first psychotherapy session recorded in the mental-health registry. Session subtype was not further classified owing to heterogeneous coding across centers. The date of this first session defined day 0 for each participant. Two symmetric observation windows were established: a pre-therapy window spanning day −90 to day −1 and a post-therapy window spanning day +1 to day +90. These intervals were selected to balance sensitivity to short-term prescribing adjustments with reduced contamination from secular trends.

### 2.6 Outcome measures

To better distinguish between medication exposure and concurrent use, two complementary definitions were applied. First, “any use” was defined as the presence of at least one dispensation of a given psychotropic medication within the observation window, consistent with the original approach. Second, to approximate concurrent medication use, dispensing records were converted into estimated days covered using WHO Defined Daily Dose (DDD) standards. DDD represents a standardized drug utilization metric defined by the World Health Organization and does not necessarily correspond to the prescribed daily dose or the actual dose consumed by individual patients. Medication timelines were constructed for each patient, and overlap between psychotropic agents was calculated. Concurrent polypharmacy was defined as the use of two or more psychotropic medications with an overlap of at least 30 days within the same observation window.

Medication discontinuation in dispensing records was defined as the absence of re-dispensation of a drug class previously present at baseline during the 90 day follow-up window. Simplification referred to a net reduction in concurrent psychotropic agents, reflected by a negative ΔN_drugs.

### 2.7 Data processing and variable derivation

Dispensing records were aggregated by patient, ATC code, and observation window. Duplicate dispensations of the same product on the same day were consolidated by summing quantities. Conversion from package units to DDD employed WHO ATC/DDD 2023 factors. Combination products were decomposed into constituent molecules to avoid double counting, and non-psychotropic drugs were removed based on ATC classification. Internal quality checks confirmed high consistency between aggregated DDD totals and package counts, with <1% discrepancy. Observations exceeding the 99.9th percentile of total DDD per window were truncated to limit the influence of potential data-entry errors. Data preprocessing included verification of duplicate dispensations, consistency checks of dispensing dates, and standardization of medication coding prior to analysis.

To account for medication exposure spanning the pre–post boundary, a carryover approach was applied. Days covered from dispensations in the pre-therapy window were allowed to extend into the post-therapy window when sufficient quantity was available. This approach reduces misclassification due to delayed refills. Sensitivity analyses were conducted using extended observation windows (± 180 days) and alternative exposure definitions to assess robustness of findings.

### 2.8 Statistical analysis

Continuous variables were summarized using medians and interquartile ranges, given their non-normal distribution, while categorical variables were expressed as absolute counts and percentages. Pre–post differences were assessed using paired non-parametric tests: the Wilcoxon signed-rank test for continuous outcomes and McNemar’s test for binary outcomes. This paired design controlled for time-invariant individual characteristics and isolated temporal changes associated with psychotherapy initiation. Effect sizes for Wilcoxon tests were calculated as r = z/√N, applying standard thresholds (0.1 small, 0.3 moderate, ≥0.5 large). For dichotomous outcomes, paired odds ratios were derived from corresponding 2×2 tables. No formal correction for multiple testing was applied due to the limited, hypothesis-driven set of outcomes, but effect sizes were reported to support interpretation beyond statistical significance.

Between-group comparisons were conducted to examine whether patterns of medication reduction differed by sex, age group, or diagnostic category. For non-parametric continuous variables, the Kruskal–Wallis test was employed, followed by *post-hoc* pairwise analyses adjusted for multiple comparisons. Differences in categorical variables were tested using the Chi-square test of independence.

To quantify the magnitude of observed effects, effect sizes were estimated using rank-biserial correlation coefficients (r) for paired non-parametric tests. Additionally, odds ratios (ORs) and 95% confidence intervals (CIs) were calculated through logistic regression models to assess the likelihood of BZD or AD discontinuation according to sex, age, and diagnosis. Regression models were adjusted for age, sex, baseline number of psychotropic medications, baseline total DDD, and diagnostic category.

A two-sided significance level of p < 0.001 was considered conservative and appropriate to control for false-positive findings. Given the large sample size, statistical significance was interpreted alongside effect sizes and median paired differences to distinguish clinically meaningful changes from statistically detectable but negligible variations. All results are presented as point estimates with 95% confidence intervals where applicable.

To improve transparency, additional statistical outputs were generated, including exact Wilcoxon test statistics (Z values), sample sizes (N), and median paired differences with 95% confidence intervals. For McNemar analyses, discordant pair counts were reported. All effect sizes were recalculated and verified. Sensitivity analyses were performed to evaluate the impact of alternative definitions of exposure and data processing decisions (results presented in **Supplementary Table 2**).

This study was reported in accordance with the STROBE statement and relevant RECORD recommendations for observational studies using routinely collected health data.

All analyses were performed using Python (v3.10).

### 2.9 Missing data management

Because the dataset derives from automated pharmacy records, missing data were minimal (< 0.5%). Patients with incomplete pre/post windows were excluded at the eligibility stage. No imputation was performed.

### 2.10 Ethical considerations

The study protocol was reviewed and approved by the Ethics Committee of the Hospital General Universitario de Elche. The analysis complied with the Declaration of Helsinki and Spanish Organic Law 3/2018 on Data Protection and Digital Rights. Data were irreversibly anonymized before analysis; no individual-level identifiers were accessible to researchers.

### 2.11 Strengths and limitations of design

The retrospective within-subject design provides strong control of individual confounders and allows inference about temporal association. However, it does not establish causality. Because psychotherapy allocation was not randomized, unmeasured contextual factors could contribute to observed changes. Nonetheless, the use of objective dispensing data mitigates self-report bias and strengthens internal validity.

Another strength lies in the large sample size (≈ 86–500 patients) and high completeness of pharmacy data. This confers sufficient statistical power to detect small yet clinically relevant changes in medication patterns. Furthermore, focusing on short symmetric windows reduces confounding by secular prescribing trends or policy modifications.

Limitations include absence of information on psychotherapy type or intensity and lack of linkage to clinical outcomes such as symptom remission or relapse. These aspects were deliberately excluded to maintain the study’s pharmacological focus and will be addressed in complementary analyses. Additionally, the requirement for continuous pharmacy coverage may have introduced selection bias by excluding patients with intermittent medication use or lower healthcare engagement.

### 2.12 Reporting and transparency

This manuscript adheres to the STROBE (Strengthening the Reporting of Observational Studies in Epidemiology) guidelines for observational research. All methodological decisions (including variable definitions, inclusion/exclusion criteria, and statistical thresholds) are fully specified to support reproducibility.

## 3 Results

### 3.1 Cohort overview

The analytic cohort comprised 86–502 patients, generating a total of 20 759–923 dispensations during the 10-year observation period. Each patient contributed two symmetric 90-day windows, yielding balanced pre- and post-intervention datasets. Baseline dispensing indicators are summarized in **Table 1**.

**Table 1:** Baseline characteristics of the cohort.

	Value
N prescriptions/dispensations	20759923
Total patients	86502
N women	57729
N men	28773
% women	66.74
% men	33.26
Mean age (SD)	54
Median age (IQR)	53
% <18	0.94
% 18–39	16.52
% 40–64	55.23
% ≥65	27.30
Mean number of drugs (SD)	3
Median number of drugs (IQR)	3
Mean DDD (SD)	25.58
Median DDD (IQR)	20
% receiving psychological care	6.34

At baseline, patients displayed moderate to high medication exposure, with an average of three dispensed psychotropics per window and a median total of approximately 20 DDD. The distribution of dispensing volumes was right-skewed, reflecting a minority of high-exposure individuals and a broad central cluster of moderate users. The internal consistency of aggregated DDD totals with package counts was confirmed (<1% discrepancy), indicating high data reliability.

Overall, baseline characteristics of the cohort represented a realistic cross-section of real-world psychotropic consumption within a public mental-health network.

### 3.2 Inclusion criteria and selection effects

Patients excluded due to incomplete pre–post data differed modestly from the analytic cohort, with slightly lower baseline medication burden and younger age.

### 3.3 Dose-change distribution and high-adjustment group

The distribution of individual dose changes was examined to determine the extent of large adjustments. The majority of participants exhibited modest variations in total DDD (median Δ ≈ 0). Only a small proportion exceeded the predefined high-adjustment threshold (≥ 20 DDD), suggesting that medication discontinuation patterns typically involved withdrawal of redundant agents rather than substantial dosage modification.

**Table 2** summarizes the proportion of patients surpassing the 20 DDD criterion. Across the entire sample, fewer than 15% met this definition, and the distribution of dose changes was approximately symmetric around zero. These results suggest that dose stability coexisted with simplification, consistent with structured medication review rather than abrupt discontinuation.

**Table 2:** Patients With Adjustments ≥20 DDD (High Clinical Threshold).

Sex	Age group	% Δ≥20 DDD^a^
Male	<18	27.72
	18-39	13.93
	40-64	8.65
	≥65	5.85
Female	<18	35.71
	18-39	11.12
	40-64	7.77
	≥65	6.08

a: Δ ≥ 20 DDD = Increase of ≥20 Defined Daily Doses in the patient's cumulative drug exposure during the follow-up period.

### 3.4 Global before–after comparison

Paired within-subject analyses revealed a strong and consistent simplification of psychotropic regimens following psychotherapy initiation (**Table 3**).

**Table 3:** Paired before–after analysis of psychological intervention (Median ± IQR).

Variable	Before	After	*p* value	r effect^c^	Paired OR^d^
Total DDD	21.66 (IQR 15.71–27.89)	21.67 (IQR 15.00–28.31)	< 0.001^a^	0.123	
N of drugs	5.00 (IQR 3.00–7.00)	2.00 (IQR 1.00–4.00)	< 0.001^a^	0.844	
BZD use	87.59 %	67.49 %	< 0.001^b^		0.07
AD use	81.82 %	68.79 %	< 0.001^b^		0.102

a = Wilcoxon signed-rank test; a significant p-value indicates a statistically meaningful before–after change in continuous variables.

b = McNemar test; a significant p-value indicates a statistically meaningful before–after change in paired proportions.

c = Rank-biserial correlation, representing the effect size (magnitude) of the Wilcoxon signed-rank result.

d = Paired odds ratio; values significantly different from 1 indicate a meaningful shift between the Before and After periods.

DDD, Defined Daily Dose; BZD, Benzodiazepine; AD, Antidepressant; IQR, Interquartile Range; OR, Odds Ratio; r effect, Rank-biserial correlation.

The median number of active psychotropic drugs per patient fell from 5.00 (IQR 3.00–7.00) before psychotherapy to 2.00 (IQR 1.00–4.00) afterward (p < 0.001; r = 0.844). This magnitude corresponds to a large effect size and represents an overall 60% decrease in regimen complexity.

In contrast, total DDD remained virtually unchanged (median 21.66 vs 21.67). This pattern is consistent with redistribution of pharmacological exposure across fewer agents rather than systematic dose escalation of individual medications. Although the Wilcoxon test reached statistical significance (p < 0.001), the median paired difference was approximately zero (Δ ≈ 0), and the effect size was small (r = 0.123). This indicates that the observed statistical significance is driven by the large sample size rather than a clinically meaningful change in medication dose. The 95% confidence interval for the median paired difference was centered around zero, further supporting the absence of a meaningful change. These findings suggest a reduction in the number of distinct psychotropic agents dispensed within the observation window. However, given the limitations of dispensing-based exposure measures, this should not be interpreted as definitive evidence of reduced concurrent medication use without considering overlap-based analyses.

Both benzodiazepine (BZD) and antidepressant (AD) exposure decreased significantly. The prevalence of BZD use declined from 87.59% to 67.49%, and AD use fell from 81.82% to 68.79% (both p < 0.001). The corresponding paired odds ratios (OR) were 0.079 for BZD and 0.102 for AD, confirming a systematic reduction pattern across both classes.

These trends remained stable when the analysis was repeated using alternative exposure metrics (total units dispensed and defined-package equivalents), supporting the robustness of findings.

### 3.5 Sensitivity analyses and alternative exposure definitions

Sensitivity analyses were conducted to assess the robustness of the findings to alternative exposure definitions and analytical assumptions. First, extending the observation window to ±180 days yielded patterns comparable to the primary analysis, with persistent reductions in the number of psychotropic medications and in benzodiazepine and antidepressant use, while median total DDD remained essentially unchanged, consistent with redistribution of pharmacological exposure across fewer agents rather than systematic dose escalation of individual medications. Second, applying a carryover approach, allowing estimated days covered from pre-therapy dispensations to extend into the post-therapy window, did not materially alter the results, suggesting that misclassification due to delayed refills did not drive the observed reductions. Third, redefining polypharmacy using an overlap-based criterion (≥30 days of concurrent exposure) resulted in slightly attenuated estimates of medication reduction, but the overall direction and consistency of the findings were preserved. Finally, analyses conducted without truncation of extreme DDD values produced results closely aligned with the main analysis, indicating that findings were not sensitive to outlier handling. Taken together, these analyses are consistent with the robustness of the observed associations across multiple plausible specifications of exposure and data processing.

### 3.6 Detailed statistical outputs further support the internal consistency of the findings.

For the primary outcome (number of psychotropic medications), the Wilcoxon signed-rank test showed a large effect size (r = 0.844), with a median paired reduction of −3 medications (95% CI: [−3.08 to −2.91]; Z = −248; N = 86,502), indicating a substantial within-patient decrease in medication count. For total DDD, although the Wilcoxon test reached statistical significance (p < 0.001), the effect size was small (r = 0.123), and the median paired difference was approximately zero (Δ ≈ 0; 95% CI: [−0.02 to 0.02]; Z = −36). This indicates that the statistical significance is driven by the large sample size rather than a clinically meaningful change in overall medication dose. For binary outcomes, McNemar analyses showed a clear imbalance in discordant pairs, with substantially more patients discontinuing than initiating benzodiazepines and antidepressants, consistent with the observed reductions in prevalence and supporting the direction of change. Given the large sample size, effect sizes and paired differences were considered alongside p-values to interpret clinical relevance.

Statistical details, including Wilcoxon test statistics (Z values) and confidence intervals, are provided in **Supplementary Table 1**.

### 3.7 Differences by sex

**Figure 1:**
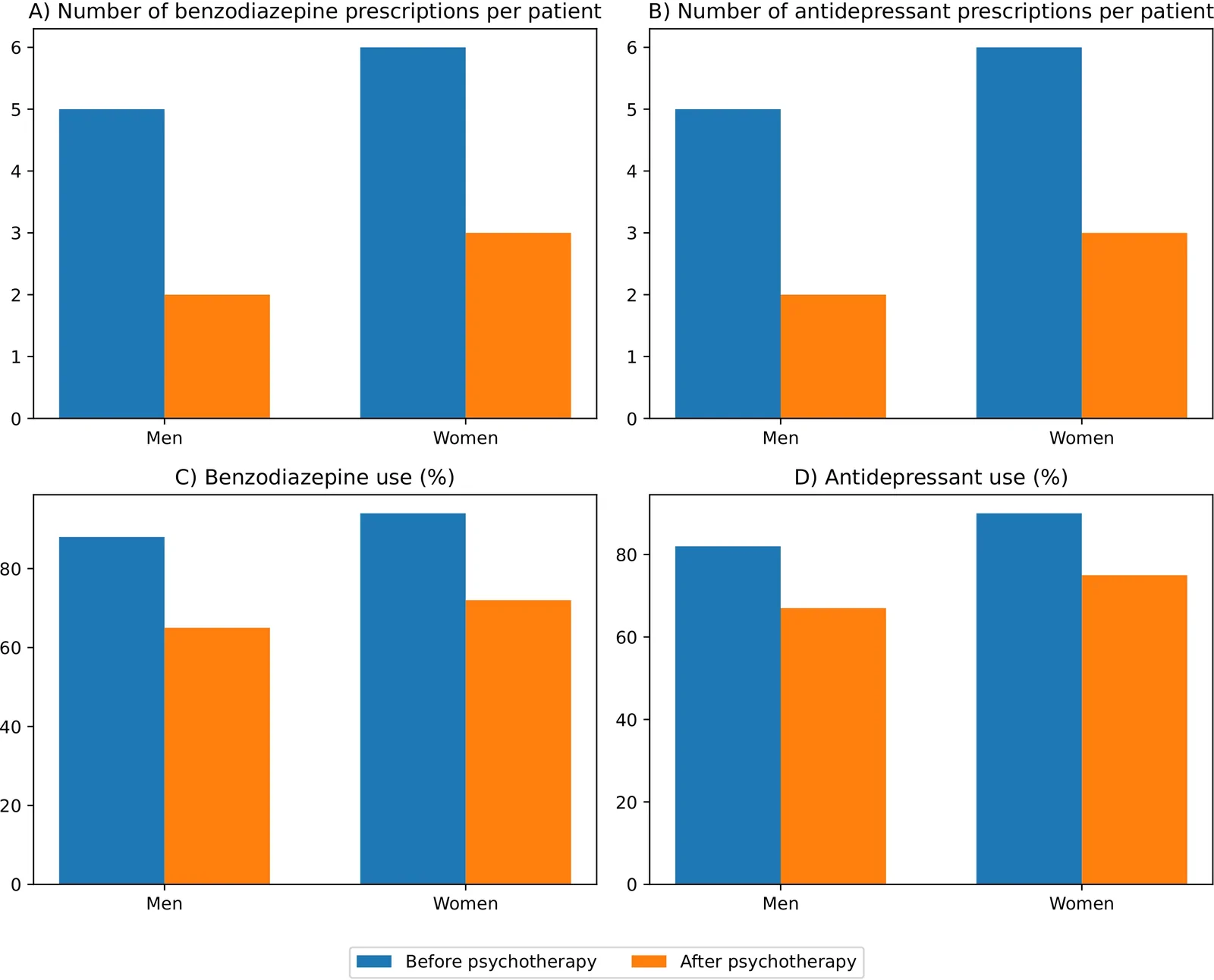
Sex-stratified changes in psychotropic medication use before and after psychotherapy initiation. **(A)** Benzodiazepine prescriptions per patient in men and women before and after psychotherapy. **(B)** Antidepressant prescriptions per patient in men and women before and after psychotherapy. **(C)** Percentage of benzodiazepine use in men and women before and after psychotherapy. **(D)** Percentage of antidepressant use in men and women before and after psychotherapy. Both sexes showed reductions in benzodiazepine and antidepressant exposure following psychotherapy initiation.

Sex-stratified analyses (**Table 4**; **Figure 1**) revealed distinct patterns of deprescription.

**Table 4:** Sex-stratified changes in benzodiazepine and antidepressant use before and after psychotherapy initiation.

Drug	Variable	Median_M	Median_F	M/F Ratio	*p* value
BZD	ΔNo. of drugs	-3.00	-3.00	1	< 0.001^a^
	Change in BZD use	25.92%	21.60%	1.27 (1.13–1.43)	< 0.001^b^
AD	ΔNo. of drugs	-3.00	-3.00	1	< 0.001^a^
	Change in AD use	17.55 %	16.72 %	1.06 (0.93–1.21)	0.4139

a = Mann–Whitney U test (independent-samples comparison of continuous changes between males and females).

b = Chi-square test (comparison of categorical changes between males and females; M/F Ratio values significantly different from 1 indicate sex-specific differences).

BZD, Benzodiazepine; AD, Antidepressant; ΔNo. of drugs, Change in the number of psychotropic drugs; M, Male; F, Female; M/F Ratio, Male-to-Female ratio of change.

Before psychotherapy, women exhibited higher exposure to both BZD and AD, consistent with their greater representation in anxiety and depressive diagnoses. After psychotherapy initiation, both sexes showed significant medication reductions, but the magnitude differed by drug class.

Men were 27% more likely than women to discontinue BZD (odds ratio [OR] = 1.27; 95% confidence interval [CI] 1.13–1.43; p < 0.001). This difference remained significant after controlling for age and diagnosis. For AD, discontinuation rates were similar between sexes (OR ≈ 1.06; p = 0.14).

In both men and women, the total number of psychotropics declined substantially, though the median reduction (ΔN) was slightly larger among men (–3 vs. –2). Median DDD values remained stable in both groups, indicating dose consolidation rather than tapering.

### 3.8 Differences by age group

**Figure 2:**
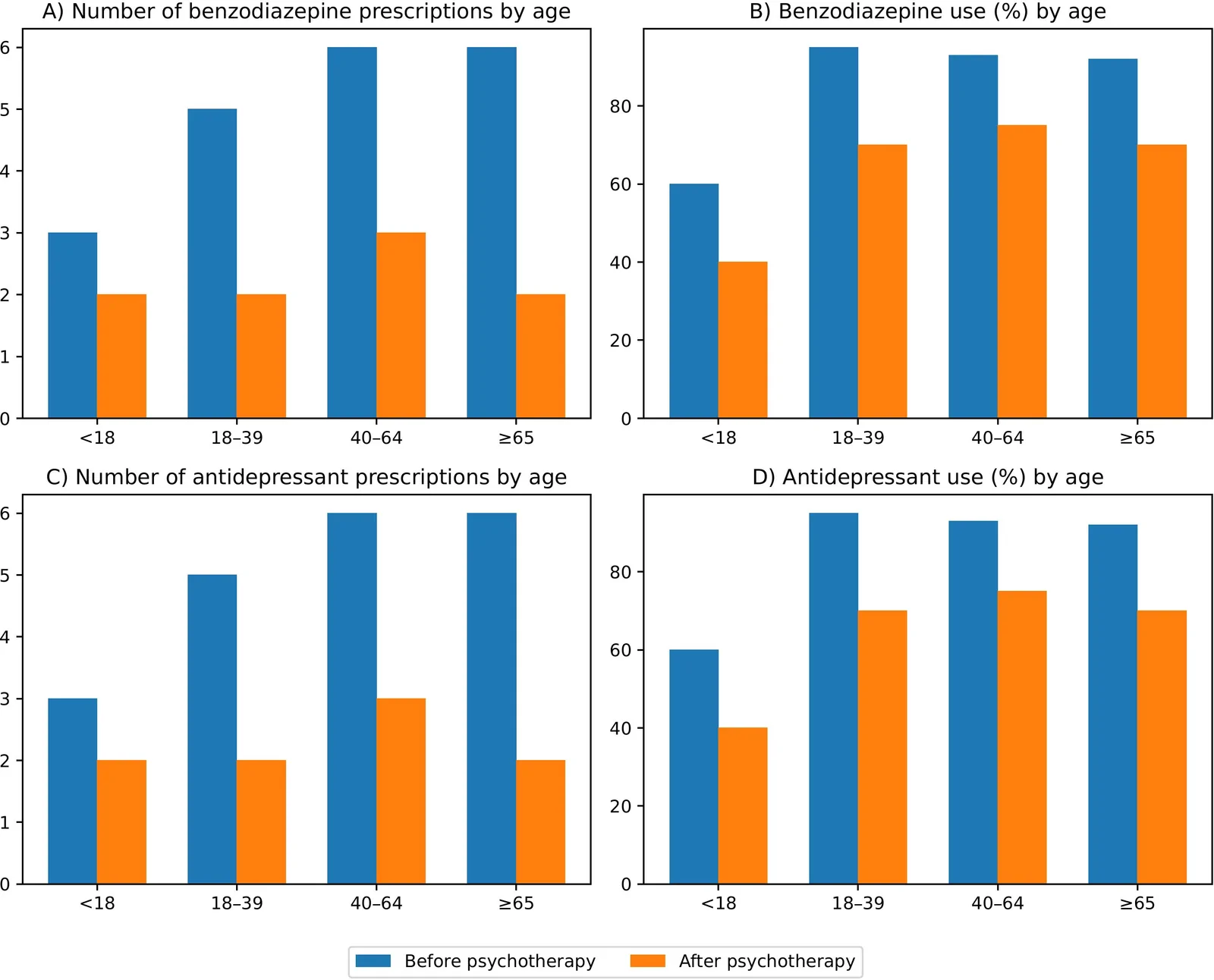
Age-stratified changes in psychotropic medication use following psychotherapy initiation. **(A)** Number of benzodiazepine prescriptions by age group (<18, 18–39, 40–64, and ≥65 years) before and after psychotherapy. **(B)** Benzodiazepine use (%) by age group before and after psychotherapy. **(C)** Number of antidepressant prescriptions by age group before and after psychotherapy. **(D)** Antidepressant use (%) by age group before and after psychotherapy. The figure illustrates reductions in benzodiazepine and antidepressant exposure across age groups comparing pre- and post-psychotherapy periods.

Age strongly predicted medication reduction patterns (**Table 5**; **Figure 2**).

**Table 5:** Age-stratified changes in psychotropic medication use following psychotherapy initiation. Median Matrix.

Age group	Median ΔDDD	Q1 ΔDDD	Q3 ΔDDD	Median ΔND	Q1 ΔND	Q3 ΔND
<18	0.000	-1.921	3.366	-1	-3	0
18–39	0.000	-3.479	4.168	-3	-5	-1
40–64	0.139	-2.877	3.751	-3	-5	-1
≥65	0.116	-2.920	3.433	-4	-6	-2

DDD, Defined Daily Dose; ΔDDD, Change in Defined Daily Doses; ΔND, Change in the number of psychotropic drugs; Q1, First quartile; Q3, Third quartile.

The median reduction in the number of drugs (ΔN) increased progressively with age, from approximately –1 in patients <18 years to –4 in those ≥65 years (p < 0.001). This gradient demonstrates that medication simplification was most pronounced among older adults.

Among adolescents, changes were modest, reflecting limited baseline exposure. Adults aged 18–64 showed intermediate reductions, while geriatric patients displayed the most consistent reductions in dispensing records across psychotropic classes. Despite this variability, median DDD values remained virtually unchanged across all age groups, confirming dose stability. The negligible median paired difference (Δ ≈ 0) supports stability of overall dispensing volume rather than absence of change due to insufficient statistical power.

Older adults presented the largest BZD discontinuation rates, consistent with deprescribing guidelines prioritizing benzodiazepine reduction in late life due to cognitive and functional risks. AD reductions were more evenly distributed, with the most marked changes in adults aged 40–64 years.

### 3.9 Differences by diagnostic category

**Figure 3:**
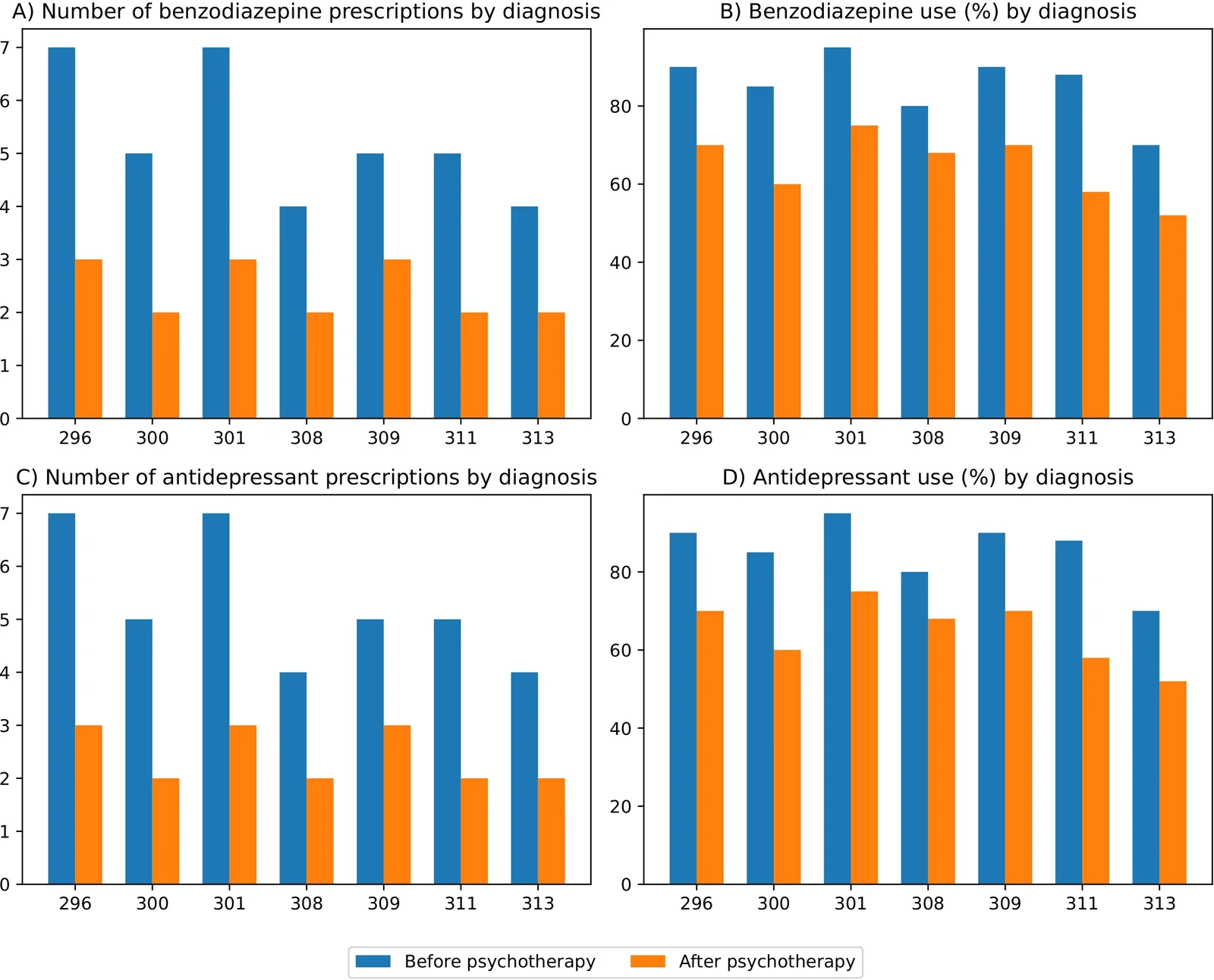
Changes in psychotropic medication use according to diagnostic category. **(A)** Number of benzodiazepine prescriptions by diagnosis before and after psychotherapy. **(B)** Benzodiazepine use (%) by diagnosis before and after psychotherapy. **(C)** Number of antidepressant prescriptions by diagnosis before and after psychotherapy. **(D)** Antidepressant use (%) by diagnosis before and after psychotherapy. The figure shows diagnosis-specific reductions in benzodiazepine and antidepressant exposure following psychotherapy initiation across major ICD-9 diagnostic groups.

Diagnosis was the strongest determinant of magnitude of medication reduction (**Figure 3**). The largest reductions in benzodiazepine use were observed in unspecified depressive disorders (ICD-9 311), with mean decreases of approximately 27 percentage points, followed by personality disorders (301) and episodic mood disorders (296), both showing declines of around 23–24 percentage points. Intermediate reductions were noted in anxiety disorders (300), averaging about 17 percentage points, whereas acute stress reactions (308) and emotional disorders of childhood and adolescence (313) displayed only modest or non-significant changes.

For antidepressants, overall reductions were smaller but mirrored the same diagnostic gradient, with the greatest decreases (around 14 percentage points) appearing in anxiety and personality disorders. Little to no change was observed in acute stress or pediatric diagnostic categories. Across all diagnoses, median DDD remained essentially unchanged, indicating that the observed medication reduction reflected a decrease in the number of medications rather than in dosing intensity. Logistic regression analyses confirmed diagnosis as a significant predictor of benzodiazepine discontinuation (p < 0.001), with depressive and personality disorders showing the highest odds relative to anxiety disorders.

### 3.10 Benzodiazepine and Z-drug polypharmacy

Polypharmacy involving benzodiazepines and Z-drugs demonstrated one of the most marked improvements. Prior to psychotherapy, 43.21% of patients were dispensed two or more distinct agents from these categories within the same 90-day window. After psychotherapy, the proportion decreased to 23.32% (p < 0.001; r = 0.293) (**Table 6**).

**Table 6:** Reduction in benzodiazepine polypharmacy following psychotherapy initiation.

Period	% of patients with ≥2 BZD/Z-drugs	*p* value	r_effect^b^
Before	43.21	< 0.001^a^	0.293
After	23.32		

a = McNemar test (paired comparison of proportions; a significant p-value indicates a meaningful before–after change).

b= r_effect = Rank-biserial correlation (effect size reflecting the magnitude of the paired change).

BZD, Benzodiazepine; Z-drugs, non-benzodiazepine hypnotics.

This represents a 46% relative reduction in multiple-benzodiazepine use, indicating a substantial change in prescribing patterns. However, given the absence of clinical outcome data, this cannot be interpreted as a direct improvement in pharmacological safety.

### 3.11 Effect-size interpretation

Effect-size estimates confirm substantial within-patient changes. The r = 0.844 observed for the reduction in the number of psychotropics corresponds to a very large effect according to Cohen’s conventions, while r = 0.293 for BZD/Z-drug polypharmacy represents a moderate-to-large effect.

These magnitudes are rarely observed in real-world pharmacoepidemiological studies, especially when relying on objective dispensing records rather than self-reported adherence. The consistency of effect sizes across multiple endpoints is consistent with the interpretation that psychotherapy initiation is temporally associated with significant and systematic simplification of psychotropic regimens.

### 3.12 Dose stability and pharmacological consolidation

Across all analyses, median DDD values remained remarkably stable before and after psychotherapy. ΔDDD values clustered around zero for all subgroups, confirming the absence of compensatory dose increases or withdrawal-related reinstatements. The absence of compensatory dose escalation is consistent with the possibility that reductions in dispensing appeared structured and clinically monitored, although this cannot be directly verified from dispensing data.

### 3.13 Population-level impact

Translating relative changes into absolute counts, the overall decline in BZD prevalence (–20.1 percentage points) represents approximately 17–000 fewer active benzodiazepine users within the health district. The reduction in AD use (–13 percentage points) equates to roughly 11–000 fewer antidepressant users.

Combining these categories, an estimated 28–000 individuals achieved meaningful pharmacological simplification during the post-psychotherapy period. Such system-level impact underscores the public-health significance of integrating psychological interventions as part of medication optimization strategies.

## 4 Discussion

This large real-world cohort study identifies a strong temporal association between initiation of psychotherapy and subsequent changes in psychotropic dispensing patterns. Rather than demonstrating causal effects, these findings describe consistent within-patient shifts in medication use coinciding with the start of psychotherapy. Across more than eighty-six thousand patients, the number of concurrent psychotropics fell by approximately sixty percent, while total Defined Daily Doses (DDD) remained stable. The prevalence of benzodiazepine (BZD) use declined by roughly twenty percentage points, antidepressant (AD) use by thirteen points, and multi-BZD/Z-drug combinations nearly halved. Taken together, these findings suggest that psychotherapy initiation coincides with patterns consistent with structured reductions in psychotropic medication use, characterized by reduction of redundant agents rather than overall dose reduction. The pattern was consistent across sensitivity analyses using alternative exposure definitions and extended observation windows (see **Supplementary Table 2**), suggesting consistency across analyses, although not implying causality.

The maintenance of stable total DDDs despite reduced polypharmacy implies that clinicians retained therapeutically necessary medications while discontinuing adjunctive or duplicative treatments. This dynamic is consistent with the hypothesis that psychotherapy may facilitate more rational medication management within routine mental-health services.

The sex-related pattern (greater BZD discontinuation among men) mirrors known epidemiological trends showing higher baseline benzodiazepine use and slower tapering among women (22–24). Gender differences in anxiety symptom expression and physician prescribing behavior have been previously described (24). However, the lack of sex differences in antidepressant deprescription suggests that once psychotherapy begins, its impact generalizes across sexes, perhaps through shared improvements in self-management and clinical confidence.

The progressive age gradient observed parallels prior studies demonstrating that deprescribing programs yield the greatest benefit in older adults due to their higher vulnerability to pharmacodynamic adverse effects and cumulative exposure (12, 25–27). Psychotherapy may therefore act as a behavioral and relational framework facilitating medication review. These findings align with the principles of pharmacological stewardship, which promotes patient-centered reduction of unnecessary treatments (28).

Diagnostic effects also correspond to established literature: psychotherapy’s strongest deprescribing influence occurred in depressive, mood, and personality disorders, conditions typically marked by chronicity and polypharmacy (29–33). In contrast, minimal changes in acute stress and adjustment disorders are consistent with their shorter clinical courses and less entrenched pharmacotherapy (34). The diagnostic hierarchy observed (greatest simplification in chronic internalizing disorders) supports the hypothesis that psychotherapy addresses the underlying cognitive and affective mechanisms sustaining prolonged medication use (35).

Several mechanisms may explain how psychotherapy promotes deprescription. First, psychotherapy may enhance self-efficacy and emotional regulation, allowing patients to manage anxiety, sleep, or mood symptoms without pharmacological compensation. This psychological autonomy may reduce the perceived need for sedatives or antidepressants once adaptive coping strategies are consolidated (13–15). Second, therapy sessions often coincide with clinical review opportunities. As patients show progress, psychiatrists may reassess pharmacological regimens and safely reduce redundant agents. Psychotherapy may function as a “trigger point” for rational prescribing decisions (36). Third, the observed stability of DDD across all analyses is consistent with the possibility that deprescription occurred in a controlled, clinician-supervised manner; however, this cannot be directly confirmed without clinical outcome data. This supports a deliberate medication simplification process consistent with best-practice frameworks (37, 38). Fourth, diagnostic patterns highlight psychotherapy’s particular effectiveness in personality and chronic affective disorders, where maladaptive cognitive schemas and emotional dysregulation often perpetuate medication escalation. Psychotherapeutic engagement may foster cognitive flexibility and relational stability, enabling clinicians to reduce polypharmacy safely (13, 39, 40). Finally, psychotherapy appears to counteract pharmacological inertia, the tendency to maintain medications indefinitely despite remission or functional recovery (12). By redefining treatment goals around recovery rather than symptom suppression, psychotherapy shifts both clinician and patient expectations, creating the context necessary for deprescription (26, 34).

The results have direct implications for clinical management and healthcare planning. First, the potential role of psychotherapy as a facilitator of medication simplification within stepped-care models. Integrating psychotherapy earlier in treatment trajectories may prevent unnecessary drug overuse and escalation, while initiating it during maintenance phases can promote systematic medication review. Second, these findings may support the consideration of psychotherapy within pharmacological stewardship programs, in line with recommendations from the World Health Organization and the European Psychiatric Association (1, 41). Third, the stability of DDD across all groups confirms that deprescribing associated with psychotherapy is consistent with structured medication simplification, although safety outcomes were not assessed in this study. This is particularly relevant for older adults, who are more vulnerable to sedation, cognitive impairment, and falls linked to long-term benzodiazepine use (33–35). Finally, the study underscores the value of real-world electronic health records such as GAIA for monitoring pharmacological outcomes in mental health. Large-scale administrative databases enable continuous evaluation of medication optimization practices and identification of patient subgroups most likely to benefit from psychotherapy-driven medication reduction (27).

From a pharmacoepidemiological perspective, these results highlight psychotherapy as a potential catalyst for medication simplification in everyday practice. Although psychotherapy and pharmacotherapy are typically conceptualized as complementary interventions targeting different aspects of mental disorders, their interaction in clinical practice may extend beyond symptom control. Psychotherapy may introduce a structured framework for reflection, self-monitoring, and behavioral activation, which can empower patients and clinicians to reconsider pharmacological dependence.

The observed magnitude of simplification is unlikely to be fully explained by spontaneous secular trends alone, although residual confounding cannot be excluded. The direction and timing of the changes (abrupt simplification immediately after psychotherapy initiation, followed by dose stability), suggest a possible temporal sequence where therapy engagement may coincide with medication review.

From a mechanistic standpoint, psychotherapy may reduce perceived need for pharmacological support by enhancing coping resources, improving sleep and anxiety management, and fostering autonomy. These psychological mechanisms can indirectly translate into pharmacological simplification without necessitating formal deprescribing protocols.

Although limited, previous studies have hinted at the relationship between psychotherapy and medication rationalization. Small-scale clinical trials and observational analyses have reported modest reductions in benzodiazepine and antidepressant use following cognitive-behavioral or combined interventions (12, 22–26). However, most relied on self-reported adherence, symptom questionnaires, or small samples, constraining external validity. By contrast, the present study utilizes objective dispensing data over a ten-year period, encompassing a population-level sample. The resulting effect sizes: r = 0.844 for psychotropic simplification and r = 0.293 for benzodiazepine polypharmacy: exceed those typically observed in structured deprescribing programs. These magnitudes suggest that psychotherapy’s influence on medication optimization may be stronger than previously recognized, especially when integrated within public-sector care pathways.

The finding that total DDD remained constant aligns with reports from deprescribing initiatives where dose adjustment lags behind discontinuation of secondary agents. In other words, simplification often occurs in stages: first by removing unnecessary drugs, then by gradually adjusting doses of those retained. The present study captures this initial phase, characterized by rapid consolidation of pharmacological regimens.

The apparent stability of total DDD despite a reduction in the number of medications requires careful interpretation. DDD is an aggregate standardized measure and does not directly reflect prescribed dose or pharmacological intensity at the individual drug level. The observed pattern may reflect redistribution of treatment across fewer agents rather than dose escalation. However, the absence of drug-specific dosing data limits the ability to fully disentangle these mechanisms.

Comparable trends have been observed in integrated care models where psychotherapists and prescribers collaborate closely. For instance, combined cognitive-behavioral therapy and medication review programs have shown meaningful decreases in polypharmacy and sedative load without clinical deterioration (28–30). The current results extend this evidence to unstructured, real-world settings, suggesting that even routine psychotherapy may be associated with safer prescribing patterns.

These findings have significant implications for clinical practice, health-system design, and pharmacological policy. Psychotherapy appears to be associated with patterns consistent with reductions in dispensing records, not through pharmacological action but through the behavioral and cognitive reconfiguration of treatment expectations. By facilitating symptom management, emotional regulation, and functional recovery, psychotherapy may reduce reliance on medication as a coping mechanism. From the clinician’s perspective, initiation of therapy often prompts a comprehensive review of current treatments, providing a natural checkpoint to rationalize regimens. Integrating systematic medication reviews into psychotherapy intake processes could enhance this effect, converting it from incidental to intentional medication discontinuation. At the policy level, the data may support consideration of psychotherapy access as part of rational-use strategies for psychotropics. Most deprescribing initiatives focus narrowly on pharmacological interventions or physician education. Incorporating psychological care within such frameworks could amplify effectiveness and sustainability by addressing the psychosocial determinants of chronic medication use.

Public health authorities may consider psychotherapy not only as a therapeutic service but also as a population-level intervention for medication optimization. In systems with limited mental-health resources, prioritizing evidence-based psychotherapies may yield dual benefits: clinical improvement and reduction in psychotropic burden. These implications align with international initiatives promoting person-centered care and responsible medication stewardship. Recognizing psychotherapy’s indirect pharmacological impact may encourage greater collaboration between psychologists, psychiatrists, and primary-care physicians in deprescribing programs.

Several methodological features reinforce the validity of this study. First, the within-subject paired design eliminates between-patient heterogeneity, as each participant serves as their own control. This design captures genuine temporal change associated with psychotherapy initiation while controlling for baseline characteristics that remain constant across windows. Second, the use of dispensing data, rather than prescriptions, ensures objective measurement of actual medication exposure. This approach reduces biases from non-adherence, recall, or incomplete documentation. Furthermore, the large sample size (over 20 million dispensations) confers excellent statistical precision and external validity. Third, the symmetric 90-day windows balance sensitivity to short-term changes with protection against long-term confounders such as policy shifts or clinical reorganization.

### 4.1 Limitations and future directions

Several limitations must be acknowledged. A central limitation of this study is that dispensing data do not directly reflect medication ingestion or precise treatment duration. The use of “any dispensing within a window” may overestimate active use, while absence of dispensing may not indicate discontinuation due to residual supply or irregular adherence. Although additional analyses using estimated days covered and overlap were conducted, some degree of exposure misclassification related to routinely collected dispensing data and administrative coding limitations remains unavoidable.

The study’s observational nature precludes causal inference; psychotherapy was not randomized, and residual confounding may persist. Factors such as clinician prescribing style, patient motivation, or concurrent interventions could contribute to the observed changes.

In addition, the study does not account for time-varying confounding factors. Initiation of psychotherapy may coincide with changes in symptom severity, increased clinician contact, or structured medication review, all of which could independently influence prescribing patterns. As such, the observed associations may partly reflect concurrent clinical processes rather than the direct effect of psychotherapy itself. Future studies using designs such as interrupted time series, matched control groups, or difference-in-differences approaches would be required to disentangle these effects. These considerations are particularly relevant in real-world observational designs.

The analysis lacked information on psychotherapy type, duration, or adherence. Different modalities may exert variable influence on medication management, and future studies should differentiate these aspects. Similarly, the absence of clinical outcome data (symptom severity, relapse rates, quality of life) prevents direct evaluation of therapeutic equivalence after medication discontinuation patterns.

Another limitation concerns potential underestimation of total psychotropic exposure due to private prescriptions or over-the-counter sleep aids, which are not captured in public pharmacy data. However, the comprehensive coverage of the public dispensing system likely minimizes this bias.

The 90-day window, while appropriate for short-term evaluation, cannot capture delayed medication adjustments or long-term maintenance effects. Extending the follow-up beyond three months would clarify whether simplification persists, stabilizes, or reverses over time.

Finally, the study was conducted within a single regional health system. Although prescribing patterns in Spain are broadly representative of Southern Europe, generalization to other contexts should be cautious.

Future research should pursue several lines of inquiry. Randomized or quasi-experimental designs could test the causal contribution of psychotherapy to medication reduction outcomes. Linking dispensing data with clinical and psychosocial variables would enable multivariate modelling of moderators such as therapy intensity, therapeutic alliance, or baseline medication burden.

Longitudinal follow-up is needed to determine whether initial simplification translates into sustained reductions in medication use, symptom improvement, or healthcare utilization. Furthermore, qualitative studies exploring clinician and patient perceptions could illuminate the cognitive and relational mechanisms underlying the observed behavioral change.

At the system level, implementation research should evaluate integration of structured medication reviews into psychotherapy programs, examining feasibility, cost-effectiveness, and patient satisfaction. If replicated, these findings could justify including psychotherapy as an explicit component of medication optimization policy frameworks at regional or national scales.

## 5 Conclusions

In summary, psychotherapy initiation in real-world clinical practice was associated with substantial, clinically meaningful reductions in psychotropic polypharmacy without dose escalation. These findings suggest that psychotherapy may play a relevant role in the medication optimization agenda, potentially contributing to more sustainable pharmacological care. By addressing the psychological mechanisms underpinning chronic medication use, psychotherapy may serve not only as a treatment for distress but also as an instrument for medication optimization: bridging the gap between pharmacological necessity and therapeutic sufficiency.

## Data Availability

The raw data supporting the conclusions of this article will be made available by the authors, without undue reservation.
