# Correction: Psychotherapy initiation is associated with discontinuation of psychotropic medications without dose escalation: a ten-year real-world cohort study (2014-2024)

**DOI:** 10.3389/fpsyt.2026.1991337

**Published:** 2026-10-08

**Authors:** Teresa Dolores Pomares Carreres, Guillermo Martínez Conesa, Francisco Navarrete

**Affiliations:** 1 Department of Mental Health, Marina Baixa Hospital, Villajoyosa, Alicante, Spain; 2 Department of Preventive Medicine and Public Health, Faculty of Medicine, University of Murcia, Murcia, Spain; 3 Instituto de Neurociencias, Universidad Miguel Hernández-CSIC, San Juan de Alicante, Alicante, Spain; 4 Red de Investigación en Atención Primaria de Adicciones, Instituto de Salud Carlos III, MICINN and FEDER, Madrid, Spain; 5 Instituto de Investigación Sanitaria y Biomédica de Alicante (ISABIAL), Alicante, Spain

**Keywords:** antidepressants, benzodiazepines, cohort study, pharmacoepidemiology, psychotherapy, psychotropic medication reduction, psychotropic polypharmacy, real-world evidence

In the **Abstract**, the sentence “The median number of psychotropic medications decreased from 5 to 2 (p < 0.001), while total dose remained stable (median Defined Daily Dose ≈ 21.7; p = 0.999).” included a p-value that, without the accompanying explanation of effect size and interpretation provided in the main text, could be misleading. This has been corrected to read:

“The median number of psychotropic medications decreased from 5 to 2 (p < 0.001), while total dose remained stable (median Defined Daily Dose ≈ 21.7).”

There was a mistake in **Figure 1** and **Figure 3** as published. These figures were inadvertently interchanged, so the figure appearing as **Figure 1** did not correspond to its caption or to the description of sex-stratified results in the main text. The corrected **Figures 1** and **3** appear below.

**Figure 1:**
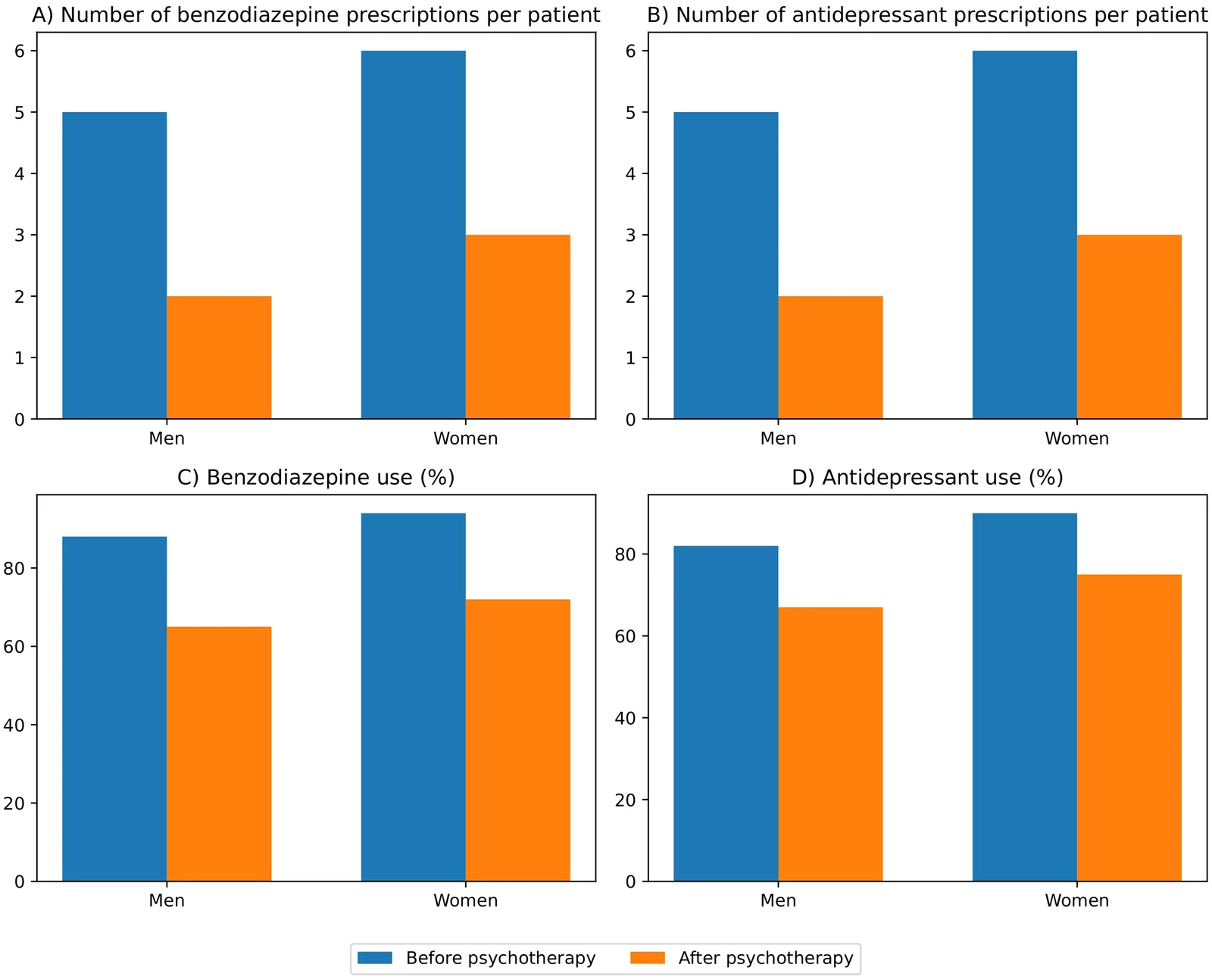
Sex-stratified changes in psychotropic medication use before and after psychotherapy initiation. **(A)** Benzodiazepine prescriptions per patient in men and women before and after psychotherapy. **(B)** Antidepressant prescriptions per patient in men and women before and after psychotherapy. **(C)** Percentage of benzodiazepine use in men and women before and after psychotherapy. **(D)** Percentage of antidepressant use in men and women before and after psychotherapy. Both sexes showed reductions in benzodiazepine and antidepressant exposure following psychotherapy initiation.

**Figure 3:**
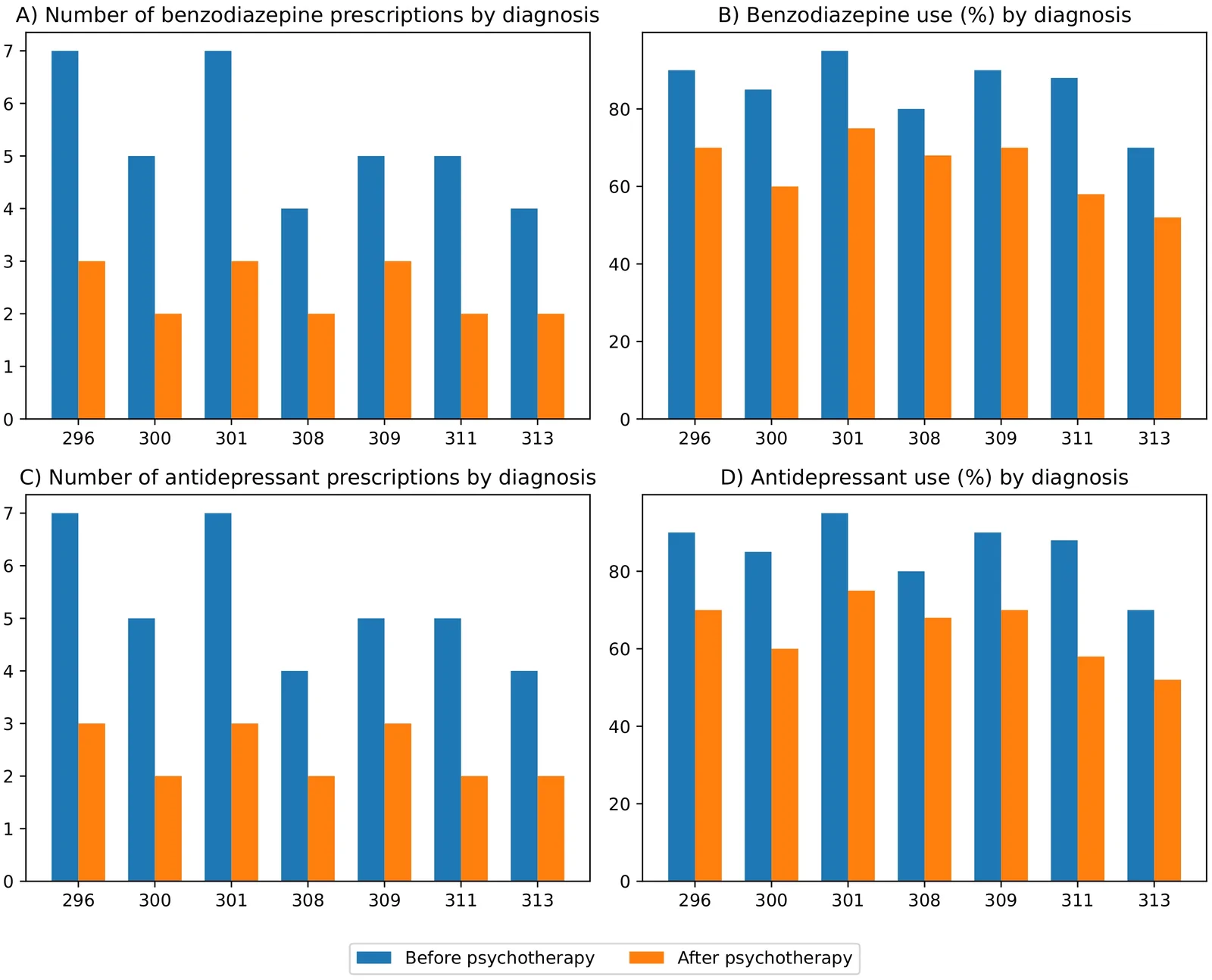
Changes in psychotropic medication use according to diagnostic category. **(A)** Number of benzodiazepine prescriptions by diagnosis before and after psychotherapy. **(B)** Benzodiazepine use (%) by diagnosis before and after psychotherapy. **(C)** Number of antidepressant prescriptions by diagnosis before and after psychotherapy. **(D)** Antidepressant use (%) by diagnosis before and after psychotherapy. The figure shows diagnosis-specific reductions in benzodiazepine and antidepressant exposure following psychotherapy initiation across major ICD-9 diagnostic groups.

The original version of this article has been updated.

