# Supplementary material for: Psychotherapy initiation is associated with discontinuation of psychotropic medications without dose escalation: a ten-year real-world cohort study (2014-2024)
Source: Front Psychiatry. 2026 Jun 22;17:1841866. doi: 10.3389/fpsyt.2026.1841866 (PMC13333713; doi:10.3389/fpsyt.2026.1841866)
Supplement: Supplementary file 1 [file Table1.docx]

**Supplementary Table S1. Detailed statistical outputs for paired before–after analyses**

| **Outcome** | **N** | **Z**ᵃ | **p-value** | **Effect size (r)**ᵇ | **Median Δ**ᶜ | **95% CI**ᵈ |
| --- | --- | --- | --- | --- | --- | --- |
| N of psychotropic medications | 86502 | -248 | <0.001 | 0.844 | -3 | [-3.08, -2.91] |
| Total DDD | 86502 | -36 | <0.001 | 0.123 | 0.00 | [-0.02, 0.02] |

a = Z values correspond to the Wilcoxon signed-rank test.

b = Effect size (r) calculated as Z / √N.

c = Median paired difference (post–pre).

d = 95% confidence interval of the paired differences.

N = number of paired observations included in each analysis.

***Abbreviations:*** *DDD = Defined Daily Dose.*
