# Supplementary material for: Psychotherapy initiation is associated with discontinuation of psychotropic medications without dose escalation: a ten-year real-world cohort study (2014-2024)
Source: Front Psychiatry. 2026 Jun 22;17:1841866. doi: 10.3389/fpsyt.2026.1841866 (PMC13333713; doi:10.3389/fpsyt.2026.1841866)
Supplement: Supplementary file 2 [file Table2.docx]

**Supplementary Table S2. Quantitative sensitivity analyses using alternative exposure definitions and observation windows**

| **Analysis** | **Variable** | **Before** | **After** | ***p* value** ^a^ | **Effect size (r)** ^b^ |
| --- | --- | --- | --- | --- | --- |
| **Main analysis** | **N of drugs** | 5.00 (IQR 3.00–7.00) | 2.00 (IQR 1.00–4.00) | <0.001 | 0.844 |
|  | **Total DDD** | 21.66 (IQR 15.71–27.89) | 21.67 (IQR 15.00–28.31) | <0.001 | 0.123 |
| **±180-day window** | **N of drugs** | 5.00 (IQR 3.00–7.00) | 2.00 (IQR 1.00–4.00) | <0.001 | 0.821 |
|  | **Total DDD** | 21.71 (IQR 15.50–28.02) | 21.74 (IQR 15.12–28.40) | <0.001 | 0.118 |
| **Carryover-adjusted exposure** | **N of drugs** | 5.00 (IQR 3.00–7.00) | 2.50 (IQR 1.00–4.00) | <0.001 | 0.731 |
|  | **Total DDD** | 21.68 (IQR 15.80–27.95) | 21.69 (IQR 15.30–28.10) | <0.001 | 0.109 |
| **Overlap-based definition (≥30 days)** | **N of drugs** | 4.00 (IQR 2.00–6.00) | 2.00 (IQR 1.00–3.00) | <0.001 | 0.612 |
|  | **Total DDD** | 20.95 (IQR 14.90–27.20) | 20.97 (IQR 14.80–27.55) | <0.001 | 0.097 |
| **No truncation of extreme DDD values** | **N of drugs** | 5.00 (IQR 3.00–7.00) | 2.00 (IQR 1.00–4.00) | <0.001 | 0.839 |
|  | **Total DDD** | 21.66 (IQR 14.10–31.85) | 21.68 (IQR 13.95–32.40) | <0.001 | 0.126 |

a = Wilcoxon signed-rank test for paired continuous variables.

b = Effect size (r) calculated as Z / √N for the Wilcoxon signed-rank test.

Despite statistical significance, median DDD values remained numerically and clinically stable across all sensitivity analyses.

***Abbreviations:*** *DDD = Defined Daily Dose; IQR = Interquartile Range.*
